# Designing a Food Hygiene Intervention in Low-Income, Peri-Urban Context of Kisumu, Kenya: Application of the Trials of Improved Practices Methodology

**DOI:** 10.4269/ajtmh.19-0629

**Published:** 2020-03-09

**Authors:** Sheillah Simiyu, Alexandra Czerniewska, Evalyne R. Aseyo, Kelly K. Baker, Oliver Cumming, Jane Awiti Odhiambo Mumma, Robert Dreibelbis

**Affiliations:** 1 African Population and Health Research Center, Nairobi, Kenya;; 2 Department of Disease Control, London School of Hygiene and Tropical Medicine, London, United Kingdom;; 3 Great Lakes University of Kisumu, Kisumu, Kenya;; 4 Department of Occupational and Environmental Health, University of Iowa, Iowa City, Iowa

## Abstract

Food contamination during weaning and complementary feeding can result in high diarrheal incidence among infants. Caregiver practices are important determinants of exposure to foodborne pathogens, and can therefore play a role in reduction in infant food contamination. Through a qualitative approach, we used the Trials of Improved Practices methodology to design a food hygiene intervention in a low-income settlement of Kisumu city in Kenya. These settlements in Kisumu city host a large portion of the city’s population and are faced with a high diarrheal disease burden. Caregivers were selected if they had a child aged 6–9 months, and together, we codesigned a combination of hardware and messaging components targeting handwashing with soap, hygienic feeding, reheating, and hygienic storage of infant food. Caregivers received up to six engagement visits with the research team. The visits were aimed at improving the designed hardware and messaging components. Results showed that feeding items were easily adopted by caregivers, whereas reheating of food was less observed. Households reportedly improved their food storage and handwashing practices. As a result, the hardware components were further refined and tested among the caregivers. Messaging components spurred the aspirations that caregivers had for their children and acted as reminders of practicing good food hygiene. The outcomes of the codesign process provided valuable insights on the knowledge of caregivers, a delivery approach for implementing the intervention, and further informed a subsequent trial that adopted the designed intervention to target early childhood exposure to enteric pathogens through contaminated food.

## INTRODUCTION

Foodborne pathogens are a contributor to diarrheal diseases, especially in young children.^1^ In low-income settings, there is a noted increase in childhood diarrheal incidence largely because of food contamination, particularly during the introduction of children to complementary and weaning foods.^1^ Contamination of food can occur anywhere between production and consumption, but caregiver practices and behaviors in the home, including hygienic preparation, storage, and feeding of weaning food, are important determinants of exposure to foodborne pathogens.^2^

A limited number of studies have described efforts to improve food hygiene behavior in the domestic environment in low- and middle-income countries. Studies from Bangladesh^3^ and Mali^4^ adopted the Hazard Analysis Critical Control Point (HACCP) approach to identify key opportunities for contamination and further implemented a health education intervention based on the identified critical points. Other studies from and Nepal^5^ and Mali^6^ applied the HACCP approach and a behavior change strategy to improve food hygiene practices. A more recent study from Malawi applied the Risk, Attitude, Norms, Ability, and Self-regulation model to assess the contextual and psychosocial factors associated with food hygiene practices.^7^ These studies have focused predominantly on rural areas.^3,5–7^ Interventions in low-income urban populations are largely limited in the literature. Furthermore, although these studies describe the interventions as delivered, they do not detail the process of intervention design, development, and pilot testing. This makes it difficult for program designers to understand how to move from epidemiological or formative research findings to a final intervention that is acceptable to target populations, feasible to deliver, and likely to result in the desired behavior change.

Codesign is a participatory approach that brings together experts and nonexperts to work together in the development of interventions.^8^ Codesign ensures that solutions are produced with an understanding of the local context and the results are acceptable to all stakeholders. Solutions that are developed from a codesign process are more acceptable to end users and more likely to be adopted and sustained.^8^ In randomized controlled trials, codesign enables community participation that results in capacity building, promotes sustained outcomes, and results in sustenance of goals beyond the funded time frames.^9^

The Trials of Improved Practices (TIPs) methodology uses a participatory approach where participants from the intended target population pilot-test candidate intervention in their own settings and provide feedback on challenges with the design or recommendations for improvement.^10^ Interventions are thus iteratively improved before introducing them on a larger scale. Trials of Improved Practices has been successfully applied to many areas of public health in low-income settings including complementary feeding programs,^11^ the design of handwashing stations,^12^ designing the feasibility of potties for child feces disposal,^13^ prolonging the life span of insecticide treated bed nets,^10^ and the development of baby play spaces.^14^

We used the TIPs methodology to design a food hygiene intervention targeting caregivers of children aged 6–9 months in low-income settlements of Kisumu city in Kenya. Our earlier research focused on infant oral contact episodes and caregiver handwashing,^15^ strategies for delivering behavior change interventions using existing health professionals,^16^ pathogen contamination of infant food,^17^ and caregiver food hygiene practices (forthcoming). These formative works showed poor hygiene practices in this setting, including frequent hand-feeding of infants, limited handwashing with soap, storage of food in inappropriate containers, and virtually no reheating of infant food.

We aimed to codesign and pilot an acceptable, feasible, and scalable intervention that included a package of household items, and accompanying messages to reduce infant’s oral exposure to fecal pathogens through improved food hygiene behaviors. Specific target behaviors identified from formative work were as follows: handwashing with soap by caregivers before food preparation and feeding, feeding children with clean utensils, hygienic child food storage, and reheating children’s food before feeding. Through a series of linked activities, we sought to identify an optimal combination of behavioral intervention components (messaging and modification to the domestic environment) to promote intervention adherence and sustainability at the household level.

### Theoretical approach.

Our approach to intervention design was informed by the behavior-centered design (BCD) theory of behavior change interventions.^18^ Behavior-centered design suggests that behavior change is most likely if an intervention can change both the “behavioral setting” and the cognitive processes associated with that behavior. The intervention makes changes in the physical, social, or biological environment of an individual, which serves as a stimulus. The surprise alters the brain of an individual, leading to a reevaluation and selection of the desired behavior, which is then rewarded.^18^ The BCD framework uses a design process and consists of an assessment of existing knowledge, building knowledge to fill the identified gaps, creating, delivering, and evaluating the intervention.^18^ The BCD approach has been used in the development and evaluation of multiple behavior change interventions.^19–21^ This study focused on the build and create stage of the BCD approach. We sought to fill knowledge gaps and build the theory of change. Previous formative work^15–17^ provided the foundation for building on knowledge in child caregiving in the settlement and building the theory of change. This knowledge was used in the create stage to develop the intervention, as reported in this article.

## MATERIALS AND METHODS

### Study site.

The study was conducted in May through September 2017 in Obunga, a peri-urban settlement in Kisumu city. Kisumu city is in Kisumu County, located in the western region of Kenya. The city is the third largest in Kenya, with a population of approximately 420,000 residents.^22^ The 2014 demographic and health survey revealed high diarrhea prevalence in the western region of Kenya (including Kisumu), of approximately 18–20%.^23^ Furthermore, the Kisumu County Integrated Development Plan highlighted diarrheal diseases as the third leading cause of morbidity among the under-fives in Kisumu County.^24^ Approximately 60% of Kisumu’s population live in informal settlements characterized by poverty, inadequate water and sanitation, and poor housing.^25^ Records from health facilities in the settlements also show that diarrheal diseases are among the top causes of morbidity.

### Study sample and recruitment.

A census of 127 children aged 3–9 months had been completed by Community Health Volunteers (CHVs) for an initial study on enteric pathogen occurrence in weaning foods.^17^ From this census list, 40 caregivers whose children were at least 6 months, and had been introduced to weaning foods were purposely identified. Caregivers were individuals who spent most of the time with the child and were involved in child food preparation and feeding. Qualitative methods were used throughout the study. These methods included focus group discussions, especially during the development of the intervention items, and in-depth interviews with caregivers during testing of the interventions at the household level.

### Ethical considerations and quality control.

The study was approved by Ethical Review Committees at the Great Lakes University of Kisumu (Ref. No. GREC/010/248/2016), at the London School of Hygiene and Tropical Medicine (Ref. No. 14695), and at the University of Iowa (IRB ID 201804204). The study was approved by the National Commission of Science and Technology and by the relevant stakeholders at Kisumu County. We sought permission from and engaged the Kisumu County Community Health Strategy focal person, the Community Health Extension workers, and the local administrative authorities including chiefs and village elders. The identified caregivers were contacted and given a written information sheet with information of the study including the objectives, their voluntary involvement, expectations, and their right to withdraw from the study. Caregivers who agreed to participate in the study gave written consent for participating in the study. Research assistants were recruited and trained on all processes of the study including ethical considerations and data collection procedures.

### Intervention development/design.

**Figure 1.**
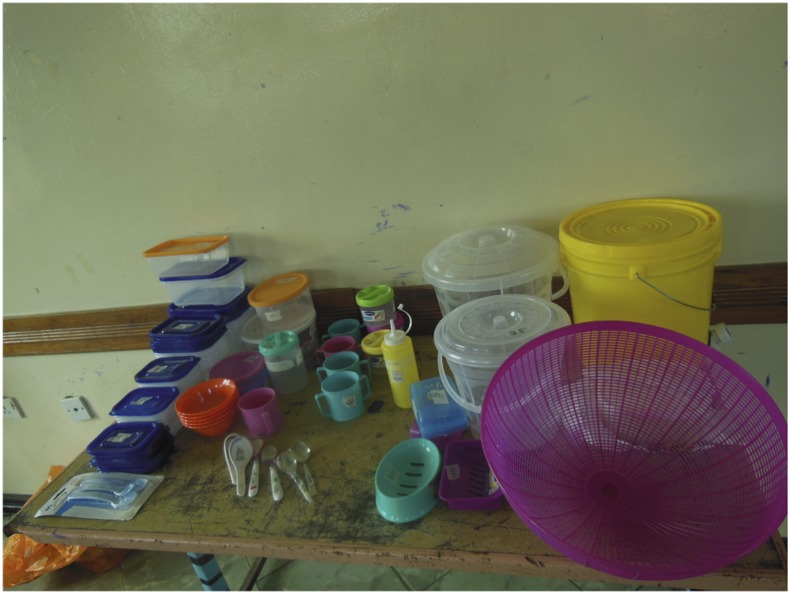
Range of items presented at a “marketplace.” This figure appears in color at www.ajtmh.org.

**Figure 2.**
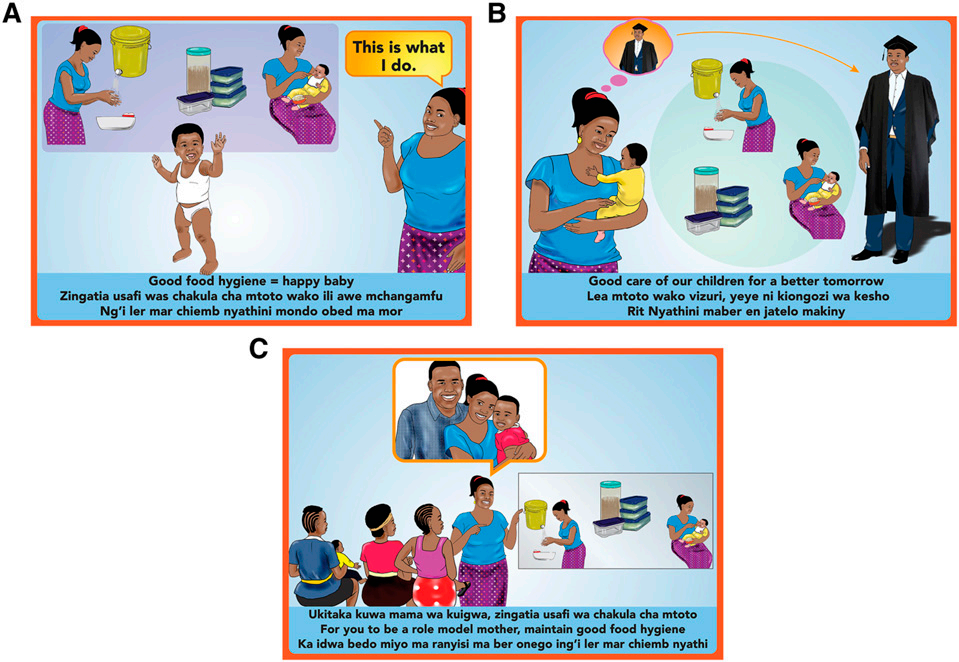
Posters showing campaign messages. (**A**) The future leader campaign, (**B**) the healthy baby campaign, and (**C**) the role model mother Campaign. This figure appears in color at www.ajtmh.org.

We started with an initial intervention package of household items (“hardware”) (Figure 1), and a behavior change communication campaign (“messaging”) (Figure 2) designed to motivate and enable uptake and adherence of the target food hygiene behaviors. This initial package was designed by our study team and refined through participatory focus group activities with caregivers. Processes for preliminary intervention design are described in Supplemental Information 1.

**Figure 3.**
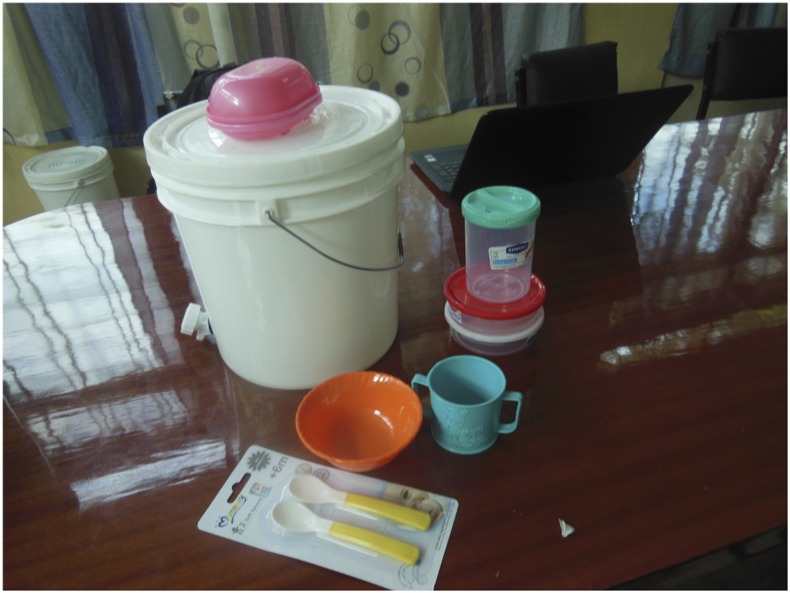
Proposed starter pack of hardware components. This figure appears in color at www.ajtmh.org.

At the start of the study, the hardware components (Figure 3) consisted of the following: 1) two shallow storage containers (for storage of solid food), 2) one deep storage container (for storage of liquid food), 3) feeding items (a bowl, cup, and pair of feeding spoons), 4) a 10-L bucket with tap (for handwashing), and 5) a soap dish.

**Figure 4.**
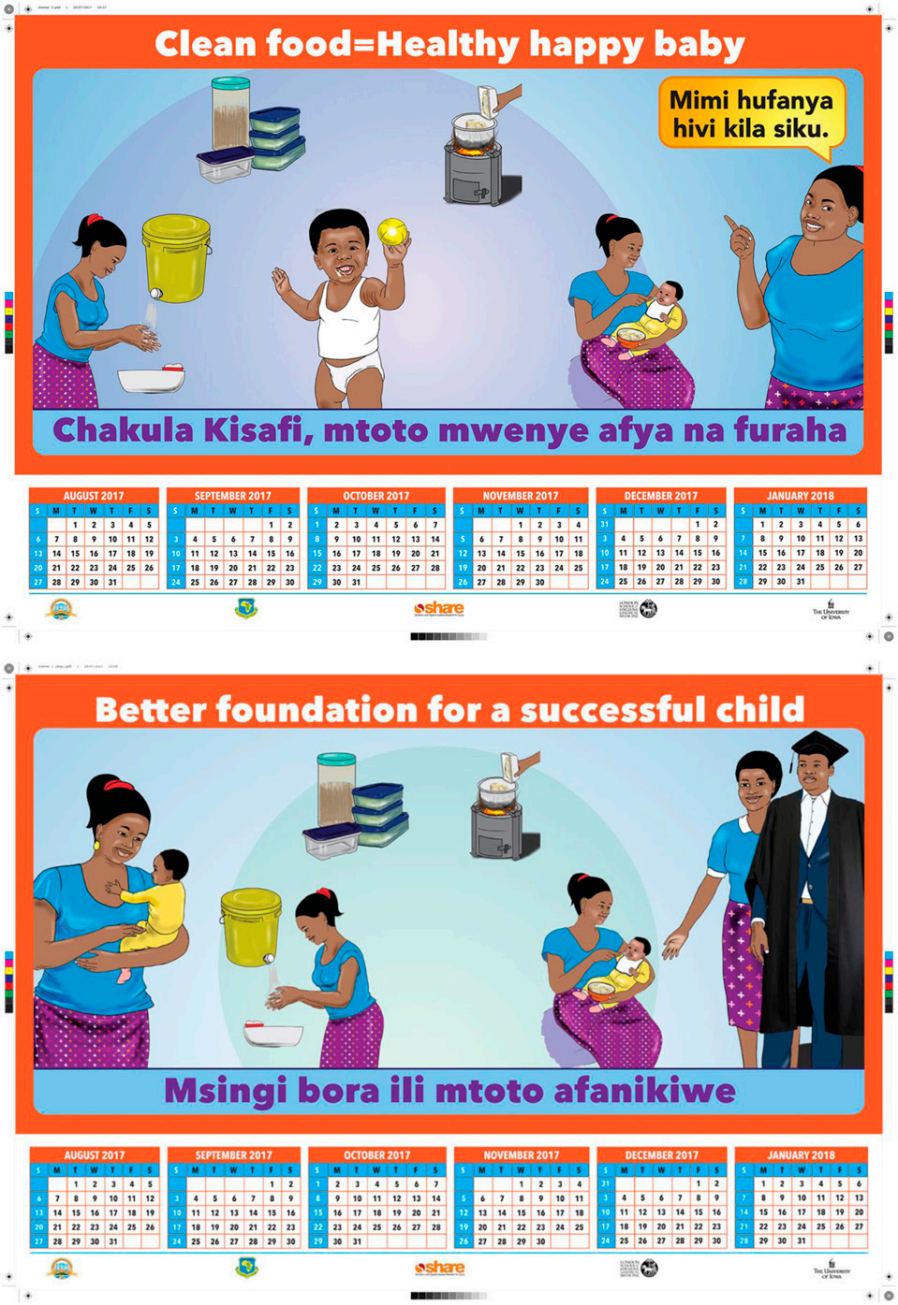
Motivational messaging campaign materials. This figure appears in color at www.ajtmh.org.

For the messaging intervention, we had two candidate campaigns with associated materials and delivery strategies: The first was “Happy Baby”—with images and messages centered on how improved food hygiene could lead to a healthy and happy child. The main message of this campaign was “Clean Food = Happy Baby” (*Chakula kisafi, mtoto mwenye furaha*). The second was “Successful Child”—with messages and images positioning food hygiene as “A Better Foundation for a Successful Child” (*Msingi bora ili mtoto afanikiwe*) (Figure 4). For both campaigns, a wall calendar and stickers (tailored for the child’s gender) with these messages were given to households, and caregivers received mobile phone text messages for 4 weeks with similar messages. The Happy Baby campaign aimed for a “playful” tone when delivered, using toy balls and other activities to entertain the children. The Successful Child campaign was more serious in tone: the mothers were asked to pledge to follow the food hygiene behaviors and then received a laminated certificate stating that “I am observing proper food hygiene so that [name of child] can be successful.”

### Codesign and testing of interventions.

The recruited participants were enrolled in the study and assigned to one of two groups. The first group of 30 caregivers was involved in the whole TIPs process (up to six visits). The second group of 10 caregivers received the intervention after iterative adaptation and modification and were only visited twice (visits 5 and 6). This second group allowed us to investigate the intervention’s acceptability and feasibility among caregivers who had not been involved in the codesign process (Table 1).

**Table 1:** Summary of visits during the trials of improved practices process

Visit	Group 1	Group 2	Total
Visit 1: Hardware delivery	30	–	30
Visit 2: Learning and feedback	26	–	26
Visit 3: Modified hardware delivery	6	–	6
Visit 4: Learning and feedback	23	–	23
Visit 5: Messaging and modified hardware	21	7	28
Visit 6: Learning and feedback	19	7	26

Interventions were delivered in teams consisting of one CHV paired with one research assistant. The CHV led the introduction process at the household level, and the research assistant led the discussions with the caregivers. Changes were made based on data collected through interviews with participants and spot-check observations. For each household, the team that led the previous visit was different from the team that led the subsequent visit to reduce courtesy bias.

At Visit 1, field teams introduced the hardware items and engaged caregivers in a discussion on how the items would be used in the household. Caregivers were asked to try and make use of the items and to pass the same message to others within their households who were involved in child caregiving.

Visit 2 occurred approximately one week later. Caregivers explained their experiences and opinions about the delivery of the hardware items, how they had used the times in the household, and reported on any challenges they had encountered. Field teams also observed if the items were in use or if there were visible indicators of recent use, and probed on the last time each of the items was used. They noted any examples of “positive deviance,” that is, where people had made alterations or adjustments to make the items easier to use. When caregivers reported challenges or had not used the items, they were encouraged to use them—for example, by setting up the handwashing bucket ready for use if it was not already in use and by explaining the benefits of preparing smaller amounts of food if the caregivers said the storage containers were too small.

Visit 3 was completed in a subset of six households 1 week after the second visit. In this visit, modified hardware items were delivered to caregivers based on feedback and observations during Visit 2. The modified items included the following: 1) two small, round, deep storage containers (similar to deep storage containers in the original pack, to aid portioning of food), 2) two small, rectangular storage containers (to replace the original shallow storage containers),3) a soap bottle filled with soapy water, and 4) a piece of bar soap.

Visit 4 was completed in all participating households 12 days after Visit 3 and followed the same methods as Visit 2 with an emphasis on discussing the use of the items, benefits gained from the use of the items, and challenges experienced.

The messaging campaigns were tested with all households at Visit 5, 8 weeks after households received the initial intervention. Half of the participating caregivers were introduced to the Happy Baby messages and half to the Successful Child messages. The field teams delivered interventions through a conversation with caregivers about how food hygiene related their child’s happiness and success, respectively. The 10 enrolled households who had not previously been involved also received the hardware items at this visit. After this visit, caregivers received text messages every 2 days for 4 weeks, at different times of the day.

Visit 6, the final visit, was completed 4 weeks later. Field teams engaged the caregivers in a discussion on the delivery and use of the hardware and messaging items. The discussion also focused on the caregivers’ interaction with the messaging items with regard to the food hygiene practices. During each of these visits, the field teams also made observations on the use of the hardware and messaging items.

## RESULTS

All caregivers reported that they felt engaged by the field team. The caregivers frequently reported that CHVs were more likely to deliver the intervention items to households they knew or their relatives, and not the caregivers who deserved them. As such, caregivers reported that they trusted the combination of the field team and the CHVs working together during delivery of the items, compared with the delivery being done by the CHVs only.

Caregivers reported using the child feeding items frequently—the spoon during feeding, the cup for feeding liquid foods, and the bowl for solid foods. According to the caregivers, the spoon, which was made of plastic, “did not hurt the baby’s gums.” Similarly, some caregivers reported that the unique color and shape of the feeding items ensured that the items were only used by the index child, and that other children were prohibited from using them. Field staff observed infant food in the bowls and cups, and in other homes, the items had been cleaned, suggesting that the feeding items were being used for their intended purposes in the home.

Storage items were used to store liquid (porridge, milk, tea, and water) and solid (potatoes, bananas, rice, and fruits) food. The items were ideal for the specified uses because “they had lids to prevent flies, dirt and dust, they could be used to store just enough amount of food [for a baby], they were portable, and could also be used for other purposes such as storing flour.” However, the original shallow storage containers were reported to become misshapen if hot food was placed in them because of the low-quality plastic, and were replaced with rectangular storage containers which caregivers indicated “were of better quality.” Caregivers reported challenges such as the small size of the storage containers, which meant that they could not store large amounts of food for an entire day, and that the containers did not keep the food warm.

The bucket was used to store water for uses such as handwashing and cleaning the children’s feeding items. It was also used by some caregivers to store feeding items for the children. Caregivers reported that the bucket “made handwashing convenient” and “was appealing due to its white color.” Challenges included leakage from the connection between the tap and bucket, lack of space to station the bucket, and difficulty in cleaning. Some caregivers improvised a stand for the bucket, most commonly a jerry can or stools in areas such as behind the door and in the cooking area. For improvement, they suggested a bigger container, with a firm tap, with smaller opening, and with tightly secured lids. These suggestions led to improvement of the handwashing container to a 20-L barrel with a tap, tested at Visit 5.

Caregivers used the soap dish for storing soap for bathing the baby rather than for handwashing as we had intended. This led us to introduce a new type of liquid soap during Visit 3. The caregivers stated that the liquid soap “made handwashing easier” and they placed it near the handwashing bucket to encourage handwashing with soap. Locally produced liquid soap was suggested as a cheaper and better option for handwashing.

With regard to messaging, although we initially thought that we had two separate candidate campaign ideas, caregivers saw it as a campaign which told a continual story. They thought the “Happy Baby” campaign was ideal when children were introduced to weaning food to motivate caregivers to focus on food hygiene, transitioning into the “Successful Child” campaign to motivate the caregivers to continue with the food hygiene practices to fulfill future aspirations for their children.

The customized calendars were hung on walls and curtains, and stickers were stuck on walls, doors, wall cabinets, and in the kitchen/cooking area. According to the caregivers, the calendars “were of good quality and did not tear easily.” Some caregivers started using the calendars as table mats instead of wall calendars.

Notably, the messages on the calendars spurred the aspirations that the caregivers had for their children. Caregivers noted that the child in the “Happy Baby” calendar looked “happy” and “healthy,” whereas caregivers who received the “Successful Child” calendar reported aspirations such as “I have a dream of my son graduating in the future” and “I want my baby to be as successful as the child in the calendar.” Caregivers reported that the messages on the calendars acted as reminders of practicing good food hygiene, but did not think that the stickers (intended to reinforce the messages by displaying it in different parts of the home) added much value. They reported that they did not learn much from the stickers because “the images were similar to those in the calendar.” Caregivers reported that the text messages were useful reminders to continue practicing good food hygiene, but that receiving text messages twice a day was “too much,” with others noting that “they did not relate with the messages” because they did not understand.

Caregivers frequently asked the field teams for more education on food hygiene and specific instruction on what they should do to maintain food hygiene. They reported that they knew hygiene was important in child care, but did not understand how their own behaviors contributed to poor food hygiene and diarrhea, for example, “I did not know that dirty food can make the baby sick” and “I did not know cooking without washing hands was harmful to the baby’s health.”

Caregivers further reported that they had changed their behaviors over the period of our visits. Observed behavioral changes confirmed storage of infant food in covered containers and separation of the baby’s items from those used by the rest of the family members. We were unable to confirm handwashing practices through observation and observed little evidence that messages increased reheating practices. A total of 14 caregivers dropped out at different stages for reasons such as travel (*n* = 6), death of the child (*n* = 1), moving out of the study area (*n* = 4), and study fatigue (*n* = 3) (Table 1). In total, 19 (of 40) caregivers completed all the six visits, seven (of 10) caregivers completed the two combined messaging and hardware visits, and 21 (of 30) households completed at least five of the six visits.

## DISCUSSION

The TIPs process described here aimed to confirm the feasibility of a designed intervention and iteratively improve the proposed package ahead of roll-out in the “Safe Start” trial (clinical trials ID NCT03468114). At the start of the TIPs process, we proposed a “starter package” combining hardware and messaging components based on the formative research findings and participatory focus group discussions. We tested this proposed intervention in households and iteratively modified messaging and hardware components through a series of household visits. The TIPs process revealed additional and often unexpected insights into the food hygiene behavior among caregivers of young children in this setting.

The use of calendars as table mats and the success with the adoption of the feeding items led to the introduction of table mats as part of the final messaging package in place of stickers. The mats served as a reminder for hygienic feeding, especially when used with the improved feeding items. This surprise result was afforded by allowing participants to experiment with intervention materials as they saw fit, a key part of the participatory process afforded by the TIPs methodology.^10^

Items that served a specific purpose in the home—such as the baby bowl and spoon–were more readily adopted by participating caregivers than items that had more general and practical applications within the household, such as buckets and soap. Caregivers valued the buckets but used them for multiple purposes in the household. Similarly, soap was used for washing clothes, dishes, and the baby, instead of for caregivers’ hands, until locally available liquid soap was introduced into the household. Setting theory posits that standing behavioral patterns within a given physical and social space are produced by the routines followed, the infrastructure and props within the physical space, and the norms and competencies of the individuals.^26^ Props such as the baby spoon and bowl were potentially more effective at “disrupting” the child feeding setting than items that served a more general purpose within the household because of their very specificity. Adapting intervention materials to target these specific junctures—such as moving away from bar soap toward liquid soap—resulted in higher rates of adoption in the household.

In designing infrastructure and props to foster and enable improved practices, other projects have explored methodologies such as human-centered design to produce locally designed and tested handwashing facilities such as the “Mrembo” or “Povu Poa” (“Cool Foam”) handwashing stations among schools in Kenya,^27,28^ and the Water and Sanitation Program’s Global Scaling Up Handwashing Project in rural Vietnam.^29^ These have highlighted the differences between settings and suggest that the effective designs are both context specific and behavior specific. Our study highlights the fact that there is still space for technological innovation in handwashing station design that meets the user’s handwashing needs while providing a specific function within the household. We accepted that our handwashing items would be used for multiple purposes but continued to include them in the intervention package because of the clear importance of provision of appropriate hardware at the household level to facilitate any behavior change.^30,31^ Compromising among the “ideal” intervention as perceived by the public health researcher, the “ideal” intervention according to the beneficiary and the viable intervention as defined by resource availability are critical in the TIPs methodology.^10^

Previous successful food hygiene interventions in Nepal used a “kitchen makeover” as a focal event in the home that facilitated the introduction of new hygiene behaviors.^5^ As in Bangladesh,^12^ we found that the very limited space in households in this peri-urban settlement limited the ability or willingness of households to designate space to a permanently erected handwashing station or have an area devoted to infant food hygiene. This in turn increased the difficulty and “friction” of performing the behavior each time and disrupted the caregivers’ ability to create a sustainable routine around handwashing.^32^ It was important to emphasize the importance of setting up the bucket for handwashing and actively encourage caregivers to plan and create designated places where the handwashing containers could be stationed. Dialog and negotiation with householders or communities have previously been found to be important in a range of low-income settings.^33–35^

Our intervention package focused on motivational messaging using emotional motivators and provision of simple but useful hardware. This was a purposeful decision, in line with the current hygiene behavior change programming, which is moving away from a focus on improving knowledge through provision of information, and toward the use of consumer marketing approaches and/or other techniques such as altering norms or strategically changing the physical environment to cue the desired actions.^36^ There is evidence from hygiene programs that knowledge of health benefits alone does not always lead to the desired change in behavior,^37^ and some interventions have shown it is possible to change hygiene behaviors without providing any health information at all.^38,39^ However, the TIPs process demonstrated that assumptions about beneficiary knowledge may not extend to food hygiene. Caregivers needed and wanted to receive information and education about food hygiene. Specifically, they knew that maintaining food hygiene was important but had not linked food hygiene to specific behaviors and practices in the home. They needed information on why and how to improve hygiene as compared with information about food hygiene. Caregivers repeated that they had learnt about the importance of food hygiene and its potential role in food contamination, despite the fact that the teams had deliberately not given a lot of information at the household visits. These results suggest that education may be a critical component of food hygiene interventions, alongside the use of other theory-driven behavior change techniques, because it increases the acceptability and likeability of the intervention.

Finally, results have highlighted the importance of building the capacity of the local community in intervention delivery. An earlier study in the same neighborhood showed the challenges facing CHVs and the need for their capacity building in hygiene interventions.^16^ We encountered a lack of confidence in CHVs, particularly in the distribution of hardware, which led us to consider a delivery method for the trial which paired CHVs with our own team members who were more trusted to deliver the products. Lack of confidence in CHVs has been reported elsewhere.^40,41^ Our findings show the importance of investigating these relationships at the start of any new intervention and forming complimentary partnerships to avoid undermining the intervention’s theory of change.

Like most TIPs processes, our study was small scale and prioritized in-depth and repeated interactions with participants over large sample sizes. We observed a high level of courtesy bias in our results, particularly in early household visits, with caregivers often reporting using the products when it was clear that they had not been used. To reduce this bias, our field team was trained to probe participants carefully, use observation to triangulate reported findings, and encourage caregivers to communicate honestly. Fostering two-way dialog between participants and data collection staff was an on-going challenge, possibly because participants were more used to receiving health education interventions than giving their opinions. The TIPs methodology generated more useful feedback on the hardware component than the messaging components of the intervention, potentially a reflection of the novelty of providing new hardware as part of a behavior change intervention. High rates of dropout with our study may have introduced selection bias in our results—caregivers who continued to engage with the intervention may have been those more who were proactive about child health and were willing to engage in the participatory process. Data from the forthcoming Safe Start trial will allow us to investigate the extent to which our intervention changed behaviors in a more general population.

## CONCLUSION

Low-income settlements present various challenges toward practicing food hygiene. Through the TIPs approach, we have described how we designed an acceptable food hygiene intervention through working with the caregivers and CHVs in the low-income settlements of Kisumu. The food hygiene intervention targeted handwashing with soap, hygienic food storage, hygienic feeding, and reheating. We designed an intervention that combined hardware items and motivational messaging items. The process led to the generation of learning points for both community participants and researchers, some of which were unforeseen at the beginning of the process. These insights informed the “Safe Start” trial that adopted the designed intervention to target early childhood exposure to enteric pathogens through contaminated food.

## Supplemental information

Supplemental materials
