# Supplementary material for: Designing a Food Hygiene Intervention in Low-Income, Peri-Urban Context of Kisumu, Kenya: Application of the Trials of Improved Practices Methodology
Source: Am J Trop Med Hyg. 2020 Mar 9;102(5):1116–23. doi: 10.4269/ajtmh.19-0629 (PMC7204591; doi:10.4269/ajtmh.19-0629)
Supplement: Supplementary file 1 [file tpmd190629.SD1.pdf]

Supplemental Information

PHASE 1: INTERVENTION DEVELOPMENT

Identification and selection of hardware items

Staff visited the local market to identify candidate household items that were affordable, readily available, and which facilitated performance of target behaviours. Identified items included food storage containers, a bucket with a spigot (for handwashing), soap dishes, cups for feeding children, hard plastic bowls for feeding children, feeding spoons, food serving spoons, and bottles for dispensing liquid soap.

Local Community Health Volunteers (CHVs) identified caregivers living within the settlement who had children aged between 6 and 9 months and were willing to participate in a focus group discussion (FGD). Two group discussions were conducted in the community, with each discussion having six participants. During the discussion, field staff clarified the roles of the participants in the discussion and answered any questions. After an initial discussion regarding food hygiene, the items that had been purchased were displayed on a table in the form of a market place, with each item having its price indicated (figure 1).

Participants were first asked to select any three items they could use within their household. Second, participants repeated the selection, but within a budget cap of 5USD. After, participants were asked to select at least one item to facilitate handwashing, one to improve food serving, and one item to improve food storage; all with a budget cap of 5USD. Following each round of selection, caregivers explained their selection, how they would use the items in their home, and provided recommendations for other items that could facilitate their own behaviour.

Results

Caregivers knew the intended purpose of all items presented. In both groups, feeding items such as baby bowls, spoons, and cups were popular among participants. Smaller storage

items were least popular with participants suggesting that the larger containers were more useful for storage, particularly for items such as flour and dry grains. The handwashing bucket was the most popular item in one group. Participants reported that the bucket had many potential uses in the households, beyond handwashing. In another group, the second group, the budgets were the least popular items; caregivers said '*they were not used to*' handwashing with buckets and preferred to use traditional basins.

After the FGDs, staff discussed the results and made suggestions of a package that could be used by caregivers for the identified hygiene practices. The proposed package consisted of a handwashing bucket and a soap dish (improving hand hygiene), a baby spoon, bowl and cup (improving feeding hygiene), and round shallow storage containers (improving food storage) (Figure 3). Although less popular than larger food storage container, smaller containers were selected because to reduce the chance that caregivers would use materials for purposes other than food hygiene. The multiple uses of the handwashing bucket in the home was used to inform subsequent messaging strategies that encouraged and motivated change in the home.

Message Development

The BCD approach guided the development of key messages for the target behaviours. We developed three campaign ideas and accompanying visual images: Campaign 1 was based on the fact that *"A healthy baby is happy, playful and feeds well*." Campaign 2 was that *"Children are seen as future leaders."* And campaign 3 that *"Maternal instincts make mothers the best caregivers."* We developed three posters with the campaign messages in three languages (English, Swahili and the local language-Dholuo) (Figure 2).

CHVs identified caregivers and invited them for a FGD to test the acceptability and suitability of the suggested motivational messages. The FGD was attended by seven caregivers who were shown the three posters, one after the other. The words were covered in order to test if

the posters were self-explanatory. Caregivers were asked to describe the message in each of the posters and to suggest key messages/words that best described the message in the posters. After this session, there was a general discussion on the message on each of the posters, after which the caregivers were asked to select their preferred posters, and to make suggestions on the most appropriate methods for delivering the messages and language of delivery.

Results

During the discussion, caregivers identified the hygiene practices and noted that the three posters told a complete and linked sequence of events in child care: experienced mothers who practiced good hygiene and had a happy family shared tips with other mothers who then took up the advice and – as a result – had children who graduated and were successful. They made further suggestions such as including a calendar on the poster, having the key messages written in English and Swahili, improving the image of the baby to illustrate a baby that is playful and happy, complementing the posters with stickers, and using mobile phone text messages.

Feedback from the FGD was consolidated and used to design the messaging strategy. A calendar would be incorporated into the poster, and the happy baby and future leader campaigns would be tested in the settlement. The future leader campaign was further renamed to 'the successful child' as it was noted that caregivers desired success for their children. The revisions were incorporated into final outputs for testing.
